# Interactions between the MicroRNAs and Microbiota in Cancer Development: Roles and Therapeutic Opportunities

**DOI:** 10.3390/cancers12040805

**Published:** 2020-03-27

**Authors:** Alessandro Allegra, Caterina Musolino, Alessandro Tonacci, Giovanni Pioggia, Sebastiano Gangemi

**Affiliations:** 1 Division of Haematology, Department of Human Pathology in Adulthood and Childhood “Gaetano Barresi”, University of Messina, 98125 Messina, Italy; cmusolino@unime.it; 2 Clinical Physiology Institute, National Research Council of Italy (IFC-CNR), 56124 Pisa, Italy; atonacci@ifc.cnr.it; 3 Institute for Biomedical Research and Innovation (IRIB), National Research Council of Italy (CNR), 98164 Messina, Italy; giovanni.pioggia@cnr.it; 4 Operative Unit of Allergy and Clinical Immunology, Department of Clinical and Experimental Medicine, University of Messina, 98125 Messina, Italy; gangemis@unime.it

**Keywords:** microbiota, miRNA, cancer, colorectal cancer, ovarian cancer, biomarkers, immune system

## Abstract

The human microbiota is made up of the fungi, bacteria, protozoa and viruses cohabiting within the human body. An altered microbiota can provoke diseases such as cancer. The mechanisms by which a modified microbiota can intervene in the onset and progression of neoplastic diseases are manifold. For instance, these include the effects on the immune system and the onset of obesity. A different mechanism seems to be constituted by the continuous and bidirectional relationships existing between microbiota and miRNAs. MiRNAs emerged as a novel group of small endogenous non-coding RNAs from that control gene expression. Several works seem to confirm the presence of a close connection between microbiota and miRNAs. Although the main literature data concern the correlations between microbiota, miRNAs and colon cancer, several researches have revealed the presence of connections with other types of tumour, including the ovarian tumour, cervical carcinoma, hepatic carcinoma, neoplastic pathologies of the central nervous system and the possible implication of the microbiota-miRNAs system on the response to the treatment of neoplastic pathologies. In this review, we summarise the physiological and pathological functions of the microbiota on cancer onset by governing miRNA production. A better knowledge of the bidirectional relationships existing between microbiota and miRNAs could provide new markers for the diagnosis, staging and monitoring of cancer and seems to be a promising approach for antagomir-guided approaches as therapeutic agents.

## 1. Introduction

### 1.1. General Considerations on Microbiota and miRNAs

The human microbiota is constituted by the fungi, bacteria, protozoa and viruses cohabiting within the human body, and it is composed by 100 trillion microorganisms [1]. Even though most researches have focused on the gut, the microbiota is not restrained to the gastrointestinal (GI) system; it also comprises the microbiota in all mucosal surfaces, the skin, genitals, nasal epithelium, and any other tissues biologically populated by microorganisms. Different body components present different microbiota. Diet, host genetics, environmental elements, stress and disease determine the configuration of the microbiota [2].

Biological equilibrium may be altered by a modified microbiota as the microbiota controls the physiological status via a regulation of metabolites, genes or proteins [3]. Recent investigations on the microbiota report its involvement in diverse diseases influencing microbiota-gut axis, microbiota–lung axis, microbiota–liver axis, microbiota–brain axis, microbiota–bone axis, microbiota–vascular axis and other axes [4]. An altered condition would provoke diseases such as cognitive impairment, allergy, autoimmunity, obesity, diabetes, inflammatory bowel disease and cancer [5,6,7].

The mechanisms by which a modified microbiota can intervene in the onset and progression of neoplastic diseases are manifold. For instance, these include the effects on the immune system and the action on the onset of obesity [8,9]. In fact, several studies have shown a correlation between obesity and the onset of malignancies. Dysbiosis might primarily cause lipid metabolism-related microRNA (miRNA) expression alteration and then provoke obesity and cancer [10].

A different mechanism seems to be represented by the continuous and bidirectional relationships existing between microbiota and miRNAs.

MiRNAs emerged as a novel group of small endogenous non-coding RNAs from 18–25 nucleotides that control gene expression via base complementarity between the seed region of the miRNA and the 3′-untranslated region (UTR) of the target mRNA. According to the level of complementarity, miRNAs bond can cause mRNA translational repression, degradation or both [11].

It is well known that miRNAs can intervene in the genesis of neoplastic disease and that their modulation could constitute an important therapeutic opportunity [12,13,14].

Several research papers seem to confirm the presence of a close connection between microbiota and miRNAs, and numerous studies executed comparative evaluation of miRNA expression of germ-free mice and animals colonised with the microbiota from pathogen-free mice. Dalmasso et al. reported nine miRNAs differently present in colonised mice relative to germ-free mice: miRNA-68, miRNA-128, miRNA-200c, miRNA-342-5p, miRNA-465c-5p, miRNA-466d-3p, miRNA-466d-5p and miRNA-665 (colon) and miRNA-298 (ileum) [15]. A similar research was performed by Singh et al. employing germ-free mice and conventionally raised mice. They reported that the microbiota influences the expression of miRNAs in the caecum [16]. Numerous putative target genes of the altered miRNAs control the synthesis of proteins implicated in the management of immune system and in the control of intestinal barrier function. Xue et al. demonstrated that the microbiota negatively controls miRNA-10 generation in intestinal epithelial cells via a MyD88-dependent pathway [17].

The relationship between microbiota and miRNAs is so close that it was possible to detect a temporal correlation between the two elements. In fact, Hicks et al. explored daily fluctuations in salivary miRNA and microbial RNA to evaluate correlations between these components. Eleven miRNAs and 11 microbial RNAs revealed constant diurnal fluctuations, while links among five circadian miRNAs and four circadian microbial RNAs were reported [18].

As mentioned above, microbiota and miRNAs seem to be able to influence each other. A deficit of Intestinal Epithelial Cells-miRNA caused gut dysbiosis and wild type (WT) faecal miRNA transplantation re-established the gut microbiota. By culturing bacteria with miRNAs, Liu et al. discovered that host miRNAs were able to modify bacterial proliferation. Oral administration of synthetic miRNA molecules is able to act on bacteria in the gut [19]. Interestingly, other authors demonstrated that animals lacking for the miRNA-producing protein, Dicer, presented an altered microbiota and were more disposed to inflammation than WT animals [20].

In a different area, Teng et al. demonstrated that exosomes-like nanoparticles (ELNs) from edible plants are taken up by gut bacteria in an ELN dependent modality, and ELN RNAs can control gut microbiota configuration [21].

**Figure 1:**
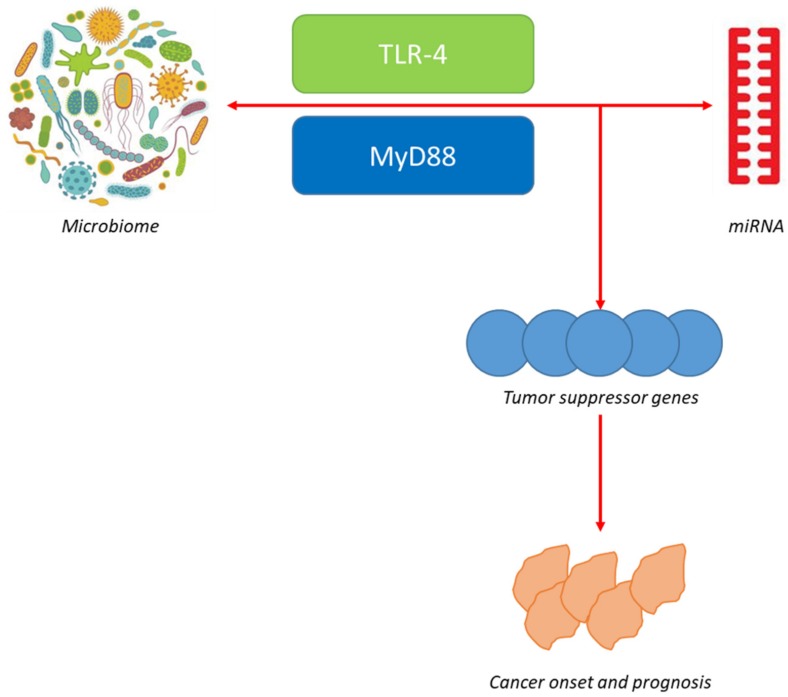
Interaction between microbiome and microRNA. An alteration of microbiome is thought to affect the regulation of various miRNAs through the MyD88-dependent pathway. miRNA alteration is, in turn, associated with gut dysbiosis; however, its re-balance would re-establish gut microbiome. The alteration of microbiome and miRNAs brings to an altered production of oncogenes and/or tumour suppressor genes, in turn affecting cancer onset and prognosis.

All these data demonstrate that faecal miRNA composition can be modified by the microbiota [22], which suggests an unknown mechanism by which the microbiota is controlled and shows that there is a chance that miRNAs could be employed therapeutically to influence the microbiota for the therapy of diseases. Moreover, these results have demonstrated the presence of a trialogue among miRNAs, microbiota and the host, and that an alteration of microbiota and miRNAs is able to provoke an altered production of oncogenes and/or tumour-suppressor genes, which can induce cancer onset and progress (Figure 1) [23,24,25].

In this review, we summarise the physiological and pathological functions of the microbiota on cancer onset by governing miRNA production.

Although the main data in the literature are related to the correlations between microbiota, miRNAs and colon cancer, several research papers have revealed the presence of connections with other types of cancer, including the ovarian tumour, cervical carcinoma, hepatic carcinoma, neoplastic pathologies of the central nervous system and the possible implication of the microbiota-miRNAs system on the response to the treatment of neoplastic pathologies.

### 1.2. Microbiota, mRNAs and Neoplastic Pathologies

#### 1.2.1. Microbiota and Ovarian Cancer

Ovarian cancer is the most fatal gynaecological tumour and the principal cause of female tumour-related death. It is characterised by the abnormal expression of miRNAs, which happens via diverse genetic and epigenetic mechanisms [26].

Numerous data seem to confirm the existence of a relationship between vaginal microbiota, miRNAs and ovarian cancer. Sierra et al. evaluated the expression of certain miRNAs after contact to bacteria-free supernatants of *Gardnella vaginalis (G. vaginalis), Lactobacillus crispatus (L. crispatus), and Lactobacillus iners (L. iners)*. Remarkably, the upregulation of miRNA-15a, miRNA-143, miRNA-145, miR-146, miR-223, and miR-148 by *G. vaginalis* bacteria-free supernatants and miR-146, miRNA-193b, and miR-223 by *L. iners* bacteria-free supernatants with no modification by *L. crispatus* reveals the existence of a bacterial species-specific modification in miRNA expression profiles [27].

Various possible mechanisms have been hypothesised to explain the relationship between microbiota and miRNAs at the vaginal level. Toll-like receptors (TLRs) are a group of pattern recognition receptors that identify microbial-derived molecules and then trigger innate immune responses [28]. They are present on both immune and cancer cells, where they modify immune response and tumour proliferation [28]. TLRs are present in several ovarian cancer cell lines and they seem to stimulate tumorigenesis by augmenting cell proliferation [29]. In ovarian cancer, TLR signalling has been connected with more aggressive pathology and worst prognosis [30]. Moreover, numerous data have correlated the TLR-4 pathway to chemo-resistance. Thus, inhibition of TLR-4 signalling may augment the effectiveness of the chemotherapy-caused programmed cell death in the ovarian cancer cells. The effects of vaginal isolated *Lactococcus lactis* on CAOV-4 cells were studied. Results revealed that *L. lactis* downregulates TLR-4, miR-21 and miR-200b expression, which relates with an increase of apoptosis. Numerous targets, including miRNA-21-5p-MKNK2, miRNA-17-5p-BCL2 and miRNA-129-5p-CDK6 were recognised, while CCNB1 and VEGFA were discovered as the hub proteins in the miRNA-target network [31].

**Figure 2:**
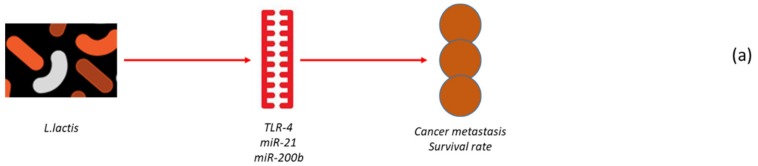

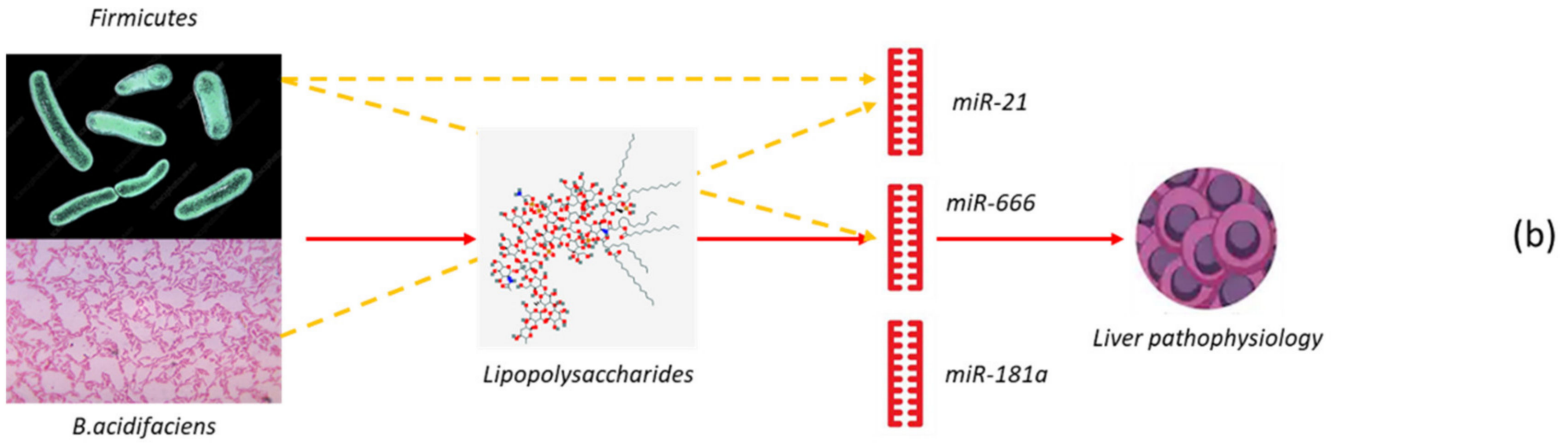
Example of the interaction between microbiome and microRNA in ovarian cancer (**a**) and hepatic carcinoma (**b**). In (**a**), *Lactococcus lactis* downregulates TLR-4, miR-21 and miR-200b (connected with cancer metastasis and survival rate) expression in CAOV4 cells, in turn leading to increased apoptosis. In (**b**), *Firmicutes* were negatively correlated with hepatic concentrations of miR-21 and miR-666, while *Bacteroides acidifaciens* was positively correlated with miR-21. As such, the expression of these miRNAs is regulated by lipopolysaccharide. In turn, miR-21, miR-666 and miR-181a are implicated in the regulation of liver pathophysiology.

These findings seem even more remarkable since several evidences demonstrated an association of the abnormal expression of miRNA-21 and miR200 family with ovarian tumour, as these miRNAs were found to be connected with cancer metastasis, and overall survival rate (Figure 2) [32,33,34]. Based on these results, the vaginal strain is able to control the ovarian cancer via a control on miRNA production, and a variation of the epigenetic action regulated by the microbiota could perhaps represent a valid clinical possibility for the prevention and therapy of the ovarian tumour.

#### 1.2.2. Microbiota and Cervical Carcinoma

Insufficient data are instead present to be able to affirm the existence of a correlation between microbiota and miRNAs in cervical carcinoma. As reported above, there is a constant communication between tumour cells and the microbiota of the vagina, and there is now emergent proof that specific types and strains that live in the normal urogenital tract defend the host against vaginal diseases and also cervical cancer [35], and a similar role could be played by some miRNAs [36].

Nonetheless, at a cervical level, the signalling pathways implicated in the microbiota-miRNAs-host connections are still inadequately recognised. However, while decreased miRNA expression is certainly not the only system influencing the defence of the cervical epithelial by microbiota, results propose that epigenetic control of cervical cell function could have a main role in safeguarding barrier integrity and protect against the malignancies [37].

#### 1.2.3. Microbiota and Hepatic Carcinoma

The microbiota may also control hepatic pathophysiology. It appears to have an action in alcohol liver disease, non-alcoholic fatty liver disease, cirrhosis and even hepatic carcinoma [37].

Recent studies demonstrated the relationship between microbiota and hepatocellular carcinogenesis [38]. The results established that sex-based disparity in liver carcinogenesis is connected with the gut microbiota and tumour-suppressive miRNAs, miRNA-22, miRNA-26a, miRNA-26a-1, miRNA-192, miRNA-122 and miRNA-125b. Microbiota controlled bile acids and miRNA production, stimulating the hepatocellular carcinoma (HCC) in a male mouse experimental model. Nevertheless, the controlling mechanism is uncertain. Increased concentrations of farnesoid X receptor (FXR), a bile acid nuclear receptor, in female animals may augment the expression of miRNA-26a, miRNA-26a-1 and miRNA-122 as the suppressors in HCC, probably causing a reduction of the risk of HCC in female mice [38]. Moreover, butyrate from microbiota increases programmed cell death via up-regulation of miRNA-22 expression and reduction of sirtuin1 expression in hepatic cells [39]. FXR has a main action in liver metabolism, liver regeneration and prevention of hepatocarcinogenesis [40]. Zhang et al. revealed that regulation of the microbiota by reduction of intestinal FXR signalling modifies liver lipid metabolism, while FXR, miRNA, and butyrate are important mediators of the microbiota involvement in HCC [41].

Moreover, the diverse phases of hepatic disorders, including HCC, are distinguishable by a specific miRNA signature, and some miRNAs, such as miRNA-21, miRNA-666 and miR-181a, are implicated in the regulation of liver pathophysiology [42].

However, it is known that the expression of these miRNAs in murine hepatocytes is regulated by lipopolysaccharide. *Firmicutes* are positively correlated and *Bacteroides acidifaciens* are negatively correlated with liver triacylglycerol concentrations. Moreover, *Firmicutes* are negatively correlated with hepatic concentrations of miRNA-21 and miRNA-666, while *B. acidifaciens* is positively correlated with miR-21. Hepatic miRNAs, liver triacylglycerols and gut microbiota could be a novel triad that can explain the mechanism by which gut microbiota regulates hepatic pathophysiology [43].

#### 1.2.4. Microbiota and the Central Nervous System

Although there are no conclusive data on the subject, it has been hypothesised that a dysregulation of the microbiota and miRNAs might be involved in the genesis of neoplastic pathologies of the central nervous system through a specific microbiota-brain axis [44]. Several findings indicate that apposite control of miRNA production within the amygdala and prefrontal cortex is regulated by microbiota structure. Several findings on mRNA targets propose miRNAs to be possibly involved in neuronal proliferation, neurogenesis and Brain Derived Neurotrophic Factor signalling. All these elements have been shown to be modified in Germ Free mice [45].

#### 1.2.5. Microbiota and Colorectal Cancer

It is certain that most of the data existing in the literature involving the relationship between microbiota, miRNAs and neoplasms are connected to the onset of the colorectal cancer (CRC)**.**

CRC is the third most frequent cause of tumour mortality and represents about 10% of all tumours overall [46].

Several research papers established that dysbiosis is a common signature of CRC. Bacteria such as *Fusobacterium nucleatum* and *Bacteroides fragilis* are constantly enriched in cancer tissues. Particular elements in those bacteria, including the FadA and Fap2 protein from *F. nucleatum* and *B. fragilis* toxins, that have a central action in CRC pathobiology, have been recognised [47,48,49].

Recent results have involved *Fusobacterium nucleatum*’s actions on the miRNome as one possible main contributor. In one study, *F. nucleatum* was demonstrated to augment CRC cell growth in mice by upregulating miRNA-21 in cancer cells by stimulating the TLR4–MyD88 signalling [50]. All this causes a reduction of the protein concentrations of RASA1 and PDCD4, both of which are cancer suppressor genes. Subjects with large quantities of *F. nucleatum* DNA and miRNA-21 showed a worse prognosis. Moreover, *F. nucleatum* has been described to increase chemoresistance to CRC by regulating autophagy in a miRNA-dependent mode [51]. Remarkably, in addition to increasing the expression of miRNA such as the miRNA-21, *F. nucleatum* caused the downregulation of miRNA-18a* and miRNA-4802, initiating TLR4–MyD88 activation. As a consequence, two central elements of the autophagy pathway, ATG7 and ULK1, which are targeted by miRNA-4802 and miRNA-18a*, were increased in CRC cells infected with *F. nucleatum*, thus avoiding undergoing chemotherapy-caused programmed cell death.

However, in CRC, miRNAs are also implicated in onset, development and metastasis [52,53,54]. Moreover, Chiang et al. asserted that miRNA-192, miRNA-194 and miRNA-215 are correlated with augmented cancer size [55], while Yuan et al. recognised 76 miRNAs as differentially expressed (DE) in CRC and normal tissues, including the oncogenic mirNA-17~92, miR-182, and miRNA-503 cluster. These DE miRNAs were associated with the abundances of numerous bacterial taxa, such as *Firmicutes, Bacteroidetes, and Proteobacteria*. Authors stated that miRNAs that correlated with CRC-associated bacteria are predicted to control targets that are central for host-microbiome relations and reported a possible action for miRNA-driven glycan production in the enrolment of pathogenic microbial taxa [56]. Several results allow to hypothesise a global relationship between microbial structure and miRNA expression in human CRC [57,58].

As mentioned above, the relationship between microbiota and miRNAs is bidirectional, and in this context, the possible action of miRNAs on the microbiota is particularly relevant. Liu et al. reported that human miRNA-515-5p could target 16S rRNA/23S rRNA of *Fusobacterium nucleatum* and that miRNA-1226-5p could modify the VegH gene of *Escherichia coli* [20].

**Figure 3:**
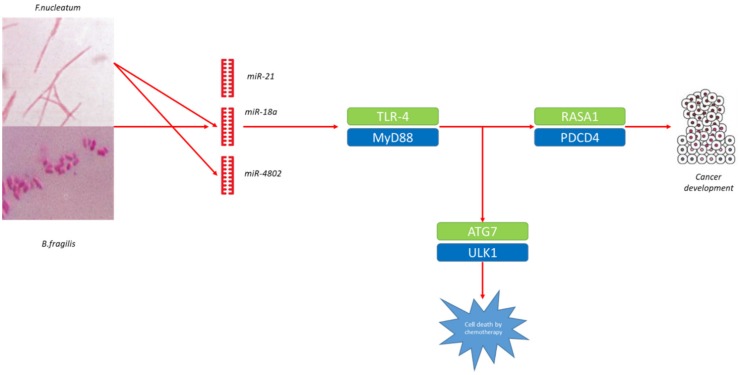
Example of the interaction between microbiome and microRNA in colorectal cancer. *Fusobacterium nucleatum* and *Bacteroides fragilis* are enriched in cancer tissues. *F. nucleatum* increases cancer cell growth by upregulating miR-21 in cancer cells by stimulating the TLR4–MyD88 signalling, in turn bringing to a reduction the protein concentrations of RASA1 and PDCD4, which are cancer suppressor genes, leading to cancer development. In addition, *F. nucleatum* downregulates miR-18a and miR-4802, initiating TLR4–MyD88 activation and increasing ATG7 and ULK1, avoiding chemotherapy-caused programmed colorectal cell death.

However, the systems by which miRNAs enter bacteria and affect specific mRNA transcription are not well-defined. In vivo researches reported that miRNAs could enter the bacteria via endocytosis [58]. Moreover, it has been demonstrated that CRC cells harbouring mutant p53 selectively discard exosomes enriched with miRNA-1246, a possible biomarker for CRC [59]. Uptake of miRNA-1246-enriched exosomes by macrophages stimulates their reprogramming into an anti-inflammatory condition, which supports cancer growth. Furthermore, faecal miRNAs, principally those originated from intestinal epithelial cells (IECs), can also control gut microbiota. MiRNA-515-5p and miR-1226-5p, which are copious in faecal samples, have been reported to support the proliferation of *E. coli* and *F. nucleatum* by entering gut bacteria and controlling their gene expression [20]. All this could create a loop capable of supporting neoplastic disease (Figure 3).

Once a correlation between microbiota, miRNAs and colorectal cancer has been established, we can attempt to analyse in detail the mechanisms by which a dysbiosis and an alteration of the non-coding genetic material can favour the onset and progression of the neoplastic disease and probably also the response to the treatment and the prognosis. It is also likely that these considerations can be widely applied to other neoplastic diseases such as those mentioned above.

Microbiota ferment non-absorbed dietary fibre to generate enormous quantities of short chain fatty acids (SCFAs). The SCFAs defend against DNA injury and mutations, which are connected with augmented tumour risk [60]. Butyrate, propionate and acetate are the most common SCFAs [61].

Butyrate offers a relevant amount of energy for normal colon epithelial cells and stimulates their growth [62]. On the other hand, butyrate causes colon tumour cell programmed death and differentiation, and reduces growth [63]. A possible mechanism of these diverse action could be a butyrate-caused transcriptional regulation through effects that reduces the expression of numerous oncogenes [64].

In fact, Butyrate has the ability to inhibit histone deacetylation (HDAC) action and thus reduce DNA injury and the action of oncogenes [65,66]. HDAC inhibitors are becoming well-known drugs for cancer treatment [67] and were initially isolated from microorganisms and continue to be developed from microbial metabolites [68].

For instance, butyrate can stimulate mRNA degradation and reduce transcript splicing. This action is able to decrease expression of c-Myc, a key proto-oncogene [69,70].

Possibly, the chemo-preventative actions of the SCFA butyrate are also provoked via the increase of p21 gene expression. Butyrate modifies the expression of 44 miRNAs in HCT-116 cells, many of which are abnormally present in colon tumour cells. Butyrate-caused p21 protein expression is inhibited by administration of a miRNA-106b mimic. Mutated p21 39UTR-reporter constructs present in HCT-116 cells proved direct miRNA-106b targeting [71].

A different study demonstrated a seven-fold increase in miRNA-92a concentrations in colon cancer cells compared to contiguous normal cells. As reported above, butyrate reduces c-Myc expression. Butyrate decreased the concentrations of pri-miRNA17-92a, precursor and mature miRNA-92a, as well as c-Myc in colon cancer cells. Mutation of the c-Myc binding site reduced butyrate’s inhibitory action on C13orf25 promoter activity. Silencing c-Myc expression decreased miRNA-92a concentrations. Conversely, c-Myc over-expression nullified butyrate-caused reduction of pri-miRNA17-92a. Exogenous miRNA-92a reduced butyrate-caused p57 expression and blocked the positive effects of butyrate on colon tumour cell growth and cell death. These results could indicate new therapeutic targets in CRC patients [72,73].

Additionally, the microbiota could produce an effect on miRNAs via an action on gene polymorphisms. One study described the connection of microbiota–related dietary elements and polymorphisms in the miRNA-binding site of the interleukin 13 gene with the incidence and the outcome of CRC. Three polymorphisms (rs1295685, rs847 and rs848) were chosen for genotyping. Two dietary elements correlated with gut microbiota (overnight meal, allium vegetables) were connected with CRC incidence [74].

Moreover, the effect of the microbiota on miRNAs and on the onset of CRC could follow other paths and be even more direct. There is new evidence that microbiota may regulate IESC growth in part via miRNAs. Peck et al. reported that miRNA signatures vary radically across different cell types of the mouse jejunal epithelium and that miRNAs react to microbiota in a cell type-specific mode. Notably, they also demonstrated that miRNAs in IESCs are more significantly controlled by microbiota with respect to any other intestinal epithelial cell type. Authors recognised miRNA-375 as one miRNA that is reduced by the microbiota in IESCs. Employing a new technique to knockdown gene and miRNA expression ex vivo enteroids, they established that it is possible to knock down gene expression in Lgr5 IESCs. Moreover, when they knock down miRNA-375 in IESCs, they noticed an augmented cell proliferation [75].

Finally, the action of microbiota and miRNAs on the genesis of CRC could take place through an effect on immunosurveillance. It has been demonstrated that T-cells can relocate miRNAs to antigen-presenting cells through exosomes, proposing that intercellular miRNA transmission might participate to regulate gene expression of the immune response [76]. SCFAs can influence differentiation and activities of T cells, dendritic cells and macrophages. Moreover, Sanchez et al. stated that at low concentrations SCFAs directly modify B cell activities, increasing class-switch DNA recombination (CSR), while reducing at higher concentrations CSR, Blimp1 and AID expression, plasma cell differentiation and somatic hypermutation. In B cells, SCFAs reduce B cell Aicda and Prdm1 by increasing specific miRNAs that target Aicda and Prdm1 mRNA-3′UTRs via a block of HDAC of those miRNA genes, impairing intestinal and systemic T-dependent and T-independent activities [77].

## 2. Conclusions

A considerable body of evidence has supported the link between cancer cell signalling, microbiota and miRNAs. From this data emerges the possibility of intervening on the onset and progression of the neoplastic disease through a modulation of the miRNAs obtained with a modification of the microbiota via the use of probiotics or a dietary modification.

For instance, CRC incidence is strictly connected to the intake of dietary types [78], and there is proof to sustain the concept that individuals who adopt Western-style diets have a superior CRC incidence than those who have a Mediterranean- or Asian-style diet [79]. It is well known that the microbiota can be influenced by diet, while miRNA expression can be regulated by dietary nutrients [80].

In an experimental human trial performed by Humphreys et al., a dietary approach was employed to evaluate the relationship between diet and miRNA-21. The dietary regimen consisted of a high red meat (HRM) intake versus a high butyrylated resistant starch (HAMSB) intake together with an HRM diet. The results demonstrated that HRM might increase miRNA-21 in human rectal mucosa. It was also reported that HRM might augment CRC incidence, while butyrate might reduce the damage caused by an HRM diet. Interestingly, concentrations of miRNA-17-92 were re-established when HRM was ingested with HAMSB, but the concentrations of miRNA-21 were not restored to the baseline [81].

Presently, the use of probiotics or diet, which intends to control microbiota, is thought as a possible therapeutic approach that has caught enormous attention. This could be an inexpensive and safe solution to re-establish health status. For instance, in addition to the possibility to modify the concentration of host-originated miRNAs, several food-originated exogenous miRNAs have been recognised. This proposes that dietary nutrients themselves are a font of miRNAs that could control homeostasis [21].

A management of the microbiota and its effects on miRNAs could also have important results in preventing damage from radiotherapy. One group of researchers has demonstrated that radiation can influence the structure of gut microbiota and miRNAs expression [82,83], while faecal microbiota transplantation from healthy subjects may present a therapeutic option for radiation-provoked toxicity [84]. Moreover, high-throughput sequencing of microbial 16S rRNA and host miRNA demonstrated that simvastatin or high-fat diet block radiation-altered enteric bacterial taxonomic structure and protect miRNA expression profile [85].

Finally, in the near future, a whole series of new research areas seem to be opening up in the context of the study on microbiota, non-coding genetic material and neoplastic disease. A fascinating field of study could be the analysis of the relationships between microbiota, neoplasms and non-coding genetic material other than miRNAs, such as long non-coding RNA (lncRNA) [86].

LncRNA expression in the gut forms a molecular signature that reveal the categories of microbes. Liang et al. confirmed the presence of a connection between lncRNA expression and gut microbes. They evidenced subgroups of lncRNAs that were distinctively enriched in each condition [87].

An RNA sequencing transcriptomic study of the *L. salivarius* strain UCC118 recognised the presence of an uncommonly abundant lncRNA encoded by the megaplasmid [88].

Bao et al. demonstrated the system by which a lncRNA plays a role in gut bacteria-provoked carcinogenesis: *Bacteroides fragilis*-associated lncRNA1 (BFAL1) in CRC cells facilitates enterotoxigenic *Bacteroides fragilis* (ETBF) carcinogenesis. BFAL1 was highly expressed in CRC cells with respect to health cells. In vitro, BFAL1 was increased in ETBF-treated CRC cells. Probably, ETBF stimulates cancer proliferation through BFAL1 by stimulating the Ras homolog, which is the MTORC1 binding/mammalian target of the rapamycin pathway. Moreover, BFAL1 controls RHEB expression by sponging miR-155-5p and miR-200a-3p. Both increased expression of BFAL1 and great quantity of ETBF in CRC cells have a bad prognostic significance for subjects with CRC [89].

The clinical relevance of the action of the microbiota on lncRNAs in the context of neoplastic diseases is confirmed by the fact that the intestinal microbiota transplant changes the host’s lncRNA expression, and this is able to diminish radiation-caused toxicity [84].

In conclusion, the study of the relationships between microbiota and miRNAs seems to be able to open countless areas of study, not only limited to neoplasms but also to other different syndromes. For instance, the possibility that miRNAs and microbiota may influence the immune response, and in particular the response mediated by T cells will need to be adequately investigated [90].

It is certain that both variables are able to influence the onset and progression of neoplasms as well as the strict dependence between them.

Further studies will allow to clarify the relationships between microbiota and miRNAs in pathologies other than those treated in this review.

For instance, lung cancer microbiota is enriched in Proteobacteria and is more diverse in adenocarcinoma and squamous cell carcinoma, particularly in males and heavier smokers [91]. On the other hand, numerous miRNAs are differently expressed in lung disease [92,93], and miRNA-21 is overexpressed in lung cancer [94], and it is well known that numerous elements of the microbiota such as *Bacteroides, Lactococcus lactis* and *Fusobacterium nucleatum* are able to modify the expression of this miRNA [31,43,50].

A deepening of the complex ratios existing between microbiota, non-coding genetic material and neoplasms could provide new markers for the diagnosis, staging and monitoring of cancer and could determine the appearance of new therapeutic approaches in the treatment of tumours, such as the use of the antagomirs as therapeutic agents.
